# Lis1 relieves cytoplasmic dynein-1 autoinhibition by acting as a molecular wedge

**DOI:** 10.1038/s41594-023-01069-6

**Published:** 2023-08-24

**Authors:** Eva P. Karasmanis, Janice M. Reimer, Agnieszka A. Kendrick, Kendrick H. V. Nguyen, Jennifer A. Rodriguez, Joey B. Truong, Indrajit Lahiri, Samara L. Reck-Peterson, Andres E. Leschziner

**Affiliations:** 1 Department of Cellular and Molecular Medicine, University of California San Diego, La Jolla, CA USA; 2 School of Biosciences, University of Sheffield, Sheffield, UK; 3 Division of Biological Sciences, Department of Cell and Developmental Biology, University of California San Diego, La Jolla, CA USA; 4 Howard Hughes Medical Institute, Chevy Chase, MD USA; 5 Division of Biological Sciences, Department of Molecular Biology, University of California San Diego, La Jolla, CA USA

**Keywords:** Cryoelectron microscopy, Dynein

## Abstract

Cytoplasmic dynein-1 transports intracellular cargo towards microtubule minus ends. Dynein is autoinhibited and undergoes conformational changes to form an active complex that consists of one or two dynein dimers, the dynactin complex, and activating adapter(s). The Lissencephaly 1 gene, *LIS1*, is genetically linked to the dynein pathway from fungi to mammals and is mutated in people with the neurodevelopmental disease lissencephaly. Lis1 is required for active dynein complexes to form, but how it enables this is unclear. Here, we present a structure of two yeast dynein motor domains with two Lis1 dimers wedged in-between. The contact sites between dynein and Lis1 in this structure, termed ‘Chi,’ are required for Lis1’s regulation of dynein in *Saccharomyces cerevisiae* in vivo and the formation of active human dynein–dynactin–activating adapter complexes in vitro. We propose that this structure represents an intermediate in dynein’s activation pathway, revealing how Lis1 relieves dynein’s autoinhibited state.

## Abstract (web summary)

Using cryo-EM, Karasmanis, Reimer, and Kendrick et al. reveal a Lis1-mediated dynein dimer, termed Chi, that serves as intermediate state in relieving dynein’s autoinhibition.

## Main

Cytoplasmic dynein-1 (dynein) is the main minus-end-directed microtubule motor responsible for transporting vesicles, protein complexes, RNAs, organelles, and viruses along microtubules. Dynein also positions nuclei and the mitotic spindle during mitosis and meiosis^1^. Dynein is conserved across eukaryotes, with the exception of flowering plants and some algae, and requires numerous binding partners and regulators to function^1,2^. Mutations in dynein or its regulators cause neurodevelopmental and neurodegenerative diseases in humans, while homozygous deletion of dynein is embryonically lethal in mice^3,4^. By contrast, in the yeast *S. cerevisiae*, dynein and its regulators are conserved but non-essential, providing an important model system to study dynein’s mechanism and function.

**Fig. 1:**
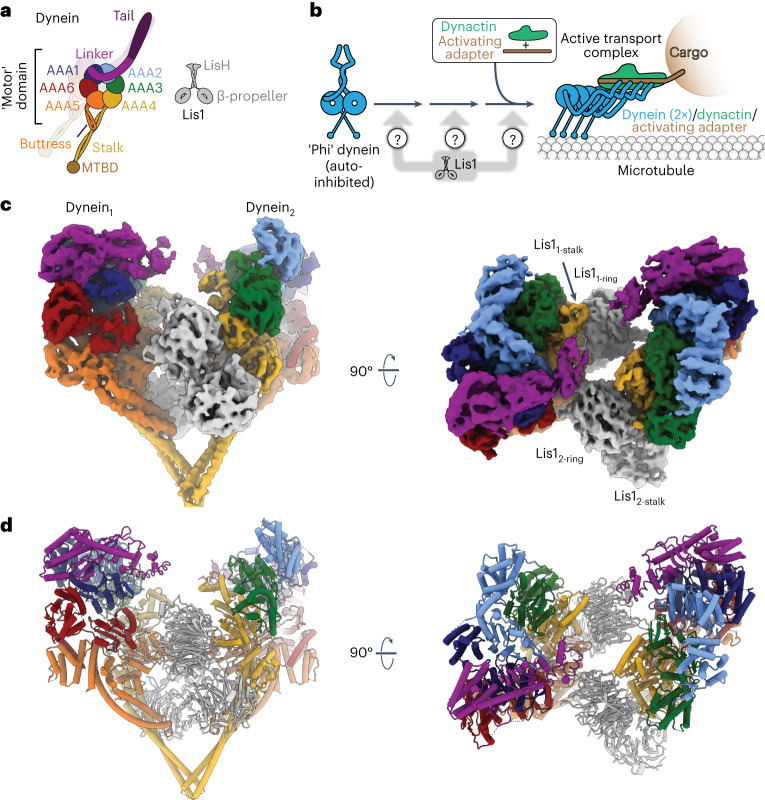
Structure of the Chi dynein–Lis1 complex. **a**, Cartoons of the domain organization of dynein and Lis1. Major elements mentioned in the text are labeled. MTBD, microtubule binding domain. **b**, Schematic of dynein activation. Activation of dynein requires the relief of its autoinhibited conformation (Phi) and the formation of an active complex containing the dynactin complex and an activating adapter. Although Lis1 is known to be involved in this process, how it is involved is unknown. **c**, Cryo-EM map of the Chi dynein–Lis1 complex (non-symmetry expanded), shown in two orientations. The different Lis1 β-propellers that bind to previously identified sites on dynein are labeled. **d**, Model of the Chi dynein–Lis1 complex, shown in the same two orientations as in the map in **c**.

Dynein is a 1.4-MDa complex consisting of a dimer of two motor-containing heavy chains composed of a ring of six AAA+ (ATPase associated with various cellular activities) domains, two intermediate chains, two light intermediate chains, and two copies of three different light chains^5^. The dynein AAA+ ring is dynamic; ATP binding and hydrolysis in AAA1 regulates dynein’s binding to and movement on microtubules. Opening and closing of dynein’s ring is coupled to movements of dynein’s mechanical element, the ‘linker,’ and rearrangements in dynein’s stalk and buttress are responsible for controlling the affinity of the microtubule-binding domain for microtubules^6^ (Fig. 1a).

Dynein is proposed to exist largely in an autoinhibited ‘Phi’ conformation in cells, which has been observed in vitro^7–9^ (Fig. 1b). In contrast to Phi dynein, which contains only the dynein subunits, active dynein complexes can be >4 MDa and consist of one or two dynein dimers, the 1.1-MDa dynactin complex, and an activating adapter that mediates the interaction between dynein and dynactin and links dynein to its cargos^10,11^ (Fig. 1b). Dynein must undergo major conformational changes to transition from the autoinhibited Phi form to these active complexes^9,12,13^. Some activated dynein complexes contain two dynein dimers, which move faster than complexes containing a single dynein dimer (Fig. 1b)^13–16^, and can include a second activating adapter^12^. Many putative activating adapters have been described, and about a dozen of these have been shown to activate dynein motility in vitro, including members of the Hook, Bicaudal D (BicD), and Ninein families^10,11,17^. Yeast dynein requires dynactin and the presumed activating adapter Num1 for function in vivo as well^18–20^.

Dynein function in vivo also requires Lis1, which is an essential positive regulator of dynein, as shown by genetic studies in many organisms^21–27^. Like dynein, Lis1 is conserved from yeast to mammals. Lis1 is a dimer, with each monomer consisting of an N-terminal small dimerization domain, an alpha helix, and a C-terminal β-propeller following a flexible linker^28,29^ (Fig. 1a). Lis1 is the only dynein regulator known to bind directly to dynein’s AAA+ motor domain, with two known binding sites: one on dynein’s motor domain between AAA3 and AAA4 (site_ring_), and the other on dynein’s stalk, a long anti-parallel coiled coil that leads to dynein’s microtubule-binding domain (site_stalk_)^30–33^. In humans, mutations in *LIS1* (*PAFAH1B1*; GeneID: 5048) or the dynein heavy chain gene (*DYNC1H1*; GeneID: 1778) cause the neurodevelopmental disease lissencephaly and other malformations of cortical development^4,34,35^. Recent studies have shown that Lis1 has a role in forming active dynein complexes^14,15,31,36^. Human dynein–dynactin–activating adapter complexes form more readily in vitro in the presence of Lis1 and move faster on microtubules owing to the recruitment of a second dynein dimer to the complex^14,15^. In *S. cerevisiae* and the filamentous fungus *Aspergillus nidulans*, mutations in dynein that block the formation of the autoinhibited Phi conformation can partially rescue Lis1 deletion or mutations, suggesting that Lis1 may activate dynein by relieving autoinhibition^21,36,37^. However, the inability of Phi-blocking mutations to fully rescue Lis1 loss-of-function phenotypes suggests that Lis1 may have additional roles in regulating dynein beyond relieving the autoinhibited Phi conformation^21,31,36^. Despite the data summarized here, how Lis1 relieves dynein autoinhibition remains unknown.

## Results

### Structure of a Lis1-mediated dynein dimer

**Extended Data Fig. 1:**
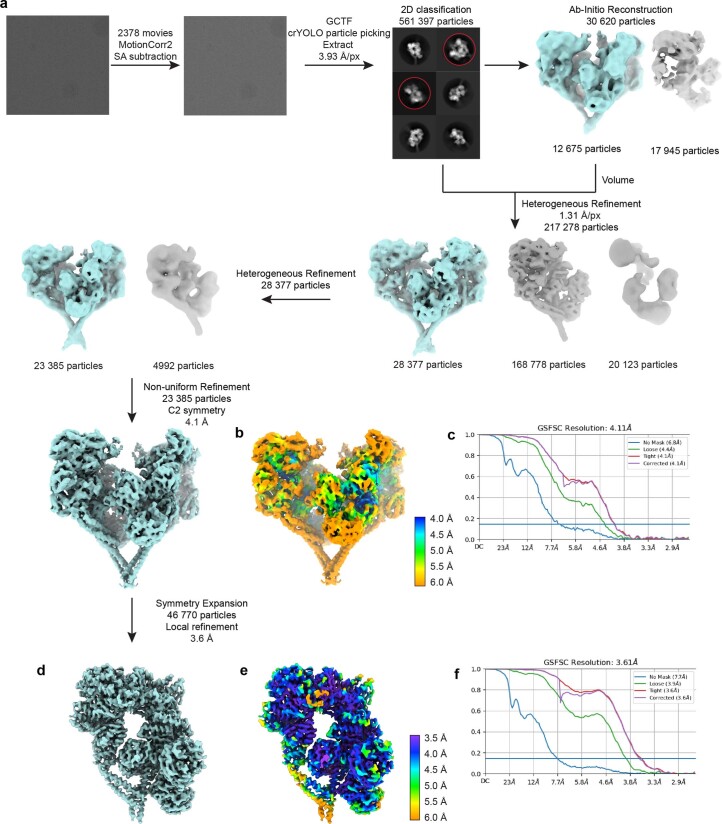
Cryo-EM data processing workflow. **a**, Processing workflow for Chi. Particles belonging to teal maps were carried into the next round of processing. **b**, Cryo-EM map of Chi (dynein^E2488Q^-Lis1)_2_ colored by local resolution. **c**, Fourier shell correlation (FSC) plot for Chi. **d**, Symmetry expansion followed by local refinement map of dynein^E2488Q^-(Lis1)_2_. **e**, Local refinement of symmetry expanded dynein^E2488Q^-(Lis1)_2_ colored by local resolution. **f**, FSC plot for local refinement map.

**Extended Data Fig. 2:**
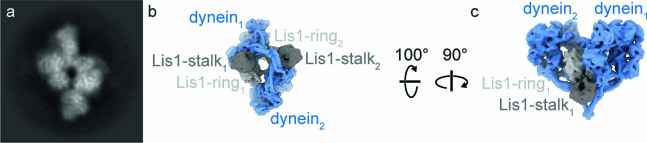
The wild-type yeast dynein motor domain can form Chi in the presence of ATP. **a**, Class average from a data set of wild-type yeast dynein motor domain incubated in the presence of Lis1 and ATP. **b**, Our model of Chi, rendered as a surface at 10 Å resolution, shown in the same orientation as the class average in (a), which corresponds to Chi being viewed from the stalks and towards the motor domains. **c**, The model in (b) is rotated to show a more canonical view of Chi. Note that Lis1-stalk_2_ appears to have partial density in the class average.

We have previously determined a high-resolution cryo-EM structure of the *S. cerevisiae* dynein motor domain (carrying an E2488Q substitution in the Walker B motif of its AAA3 domain) in the presence of ATP-vanadate and bound to two yeast Lis1 β-propellers, most likely coming from the same Lis1 dimer^31^. Although this data set appeared to consist primarily of particles containing one dynein motor domain bound to one Lis1 dimer, we revisited it to look for other structures. To our surprise, additional data processing revealed several two-dimensional (2D) class averages that contained two dynein motor domains, even though the dynein that we used for cryo-EM sample preparation was engineered to be monomeric. Using this subset of particles, we solved a structure of two dynein motor domains in complex with two Lis1 dimers to 4.1 Å. We called this structure ‘Chi’ because the dynein conformation resembles the Greek letter Chi, and because Chi follows Phi in the Greek alphabet (Fig. 1c,d, Supplementary Video 1, Extended Data Fig. 1, and Table 1). In our structure, two dynein motor domains, each bound by a Lis1 dimer, come together through dynein–Lis1 interactions which, to the best of our knowledge, have not been previously reported. This arrangement is consistent with previous studies that have reported the binding of two Lis1 dimers to dynein in solution^14,38,39^. In our Chi structure, the closed conformation of dynein’s ring and the characteristic bulge in the stalk near its contact site with the buttress indicate a weak microtubule-binding state^40^. The overall structure of each dynein–Lis1 complex is very similar to the structures that we have solved of individual dynein motor domains bound to a Lis1 dimer, also in a weak microtubule-binding state^30,31^. Importantly, the Chi conformation is not a consequence of the E2488Q substitution in dynein, or the use of ATP-vanadate in the sample: we also observed Chi in a data set collected from a sample containing wild-type dynein monomer, ATP, and Lis1 (Extended Data Fig. 2 and Table 1). The presence of only a single class average of Chi in that data set prevented us from obtaining a 3D reconstruction.

**Table 1:** Cryo-EM data collection, refinement and validation statistics

	Dynein^E2488Q^ bound to Lis1 in Chi conformation. EMD-27810, PDB 8DZZ	Dynein^E2488Q^ bound, to Lis1 in Chi, conformation, symmetry expansion, EMD-27811, PDB 8E00	Dynein bound, to Lis1 in the presence of ATP
**Data collection and processing**			
Magnification	38,000	38,000	36,000
Voltage (kV)	300	300	200
Electron exposure (e^–^/Å^2^)	58.3	58.3	51.0
Defocus range (μm)	2–2.7	2–2.7	0.8–2.4
Pixel size (Å)	1.31	1.31	1.16
Symmetry imposed	*C*_2_	*C*_1_	
Initial particle images (no.)	561,397	561,397	
Final particle images (no.)	23,385	46,770	
Map resolution (Å)	4.1	3.6	
FSC threshold	0.143	0.143	
Map resolution range (Å)	4.0–6.0	3.5–6.0	
**Refinement**			
Initial model used (PDB code)	8E00	7MGM	
Model resolution (Å)	4.4	3.9	
FSC threshold	0.5	0.5	
Map sharpening *B* factor (Å^2^)	106.5	90.5	
Model composition			
Non-hydrogen atoms	49,596	24,794	
Protein residues	6,110	3,055	
Ligands	8	4	
*B* factors (Å^2^)			
Protein	154.6	94.0	
Ligand	132.4	77.6	
R.m.s. deviations			
Bond lengths (Å)	0.005	0.005	
Bond angles (°)	1.087	1.124	
Validation			
MolProbity score	1.11	0.98	
Clashscore	3.00	2.10	
Poor rotamers (%)	0.25	0.47	
Ramachandran plot			
Favored (%)	98.39	98.21	
Allowed (%)	1.57	1.76	
Disallowed (%)	0.03	0.03	

**Fig. 2:**
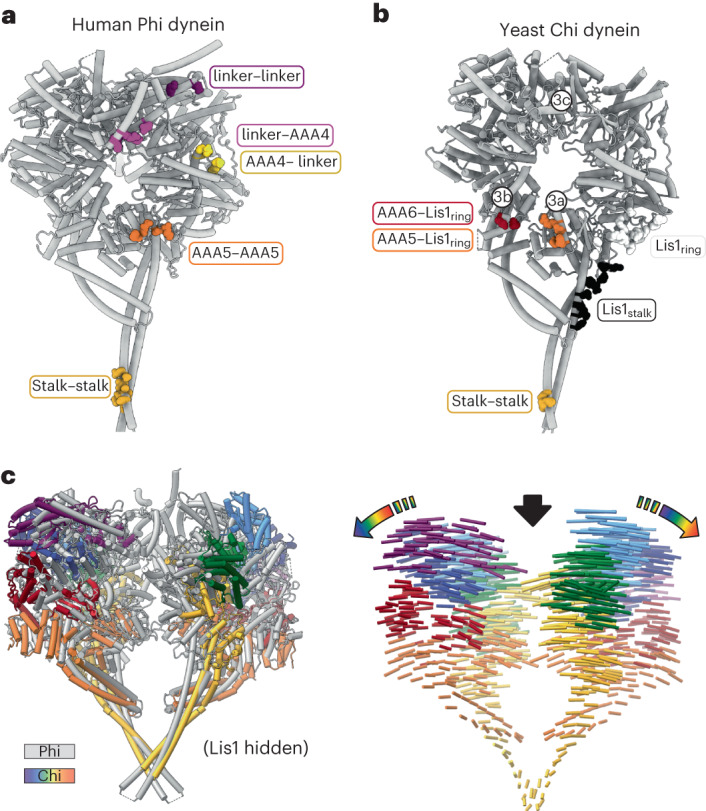
Comparison of dynein in Chi and Phi. **a**, Interfaces in human dynein involved in stabilizing Phi (PDB: 5NUG)^9^. **b**, Interfaces in yeast dynein involved in stabilizing Chi. Note that all but one (stalk–stalk) of the interfaces in Chi also involve Lis1, which we do not show in this panel. The circled labels (3a, 3b, 3c) refer to panels in Figure 3, in which interfaces are shown in detail. **c**, Overlay of dynein in Phi (gray) and Chi (rainbow). As in panel (b), we omitted Lis1 in the Chi structure for clarity. The left panel shows the two structures superimposed. The right panel shows interatomic vectors linking equivalent alpha carbons in Phi and Chi. The length of the cylinders is proportional to the displacement of that atom between the two structures.

**Extended Data Fig. 3:**
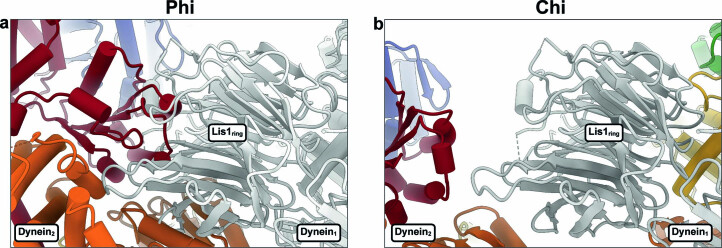
Binding of Lis1 to dynein is sterically incompatible with Phi. **a**, Phi dynein (PDB 5NUG) was aligned to dynein1 in Chi, which is shown in gray in this panel. Phi’s dynein_2_, shown in color, clashes with the Lis1 bound at site_ring_ in Chi’s dynein_1_. **b**, Close up of our model of Chi equivalent to the view in (a), showing Lis1 bound at site_ring_ on dynein_1_. Part of dynein_2_, with which the Lis1 at site_ring_ interacts in Chi, is also shown.

Dynein’s autoinhibited Phi conformation is characterized by interactions between its two motor domains that make them point in opposite directions (they are effectively ‘cross-legged’) (Figs. 1b and 2a), which prevents the motor from being able to bind to microtubules^9^. The Phi conformation is incompatible with the binding of Lis1 to dynein, as Lis1 bound to one dynein clashes with the second dynein in Phi (Extended Data Fig. 3a).

**Fig. 3:**
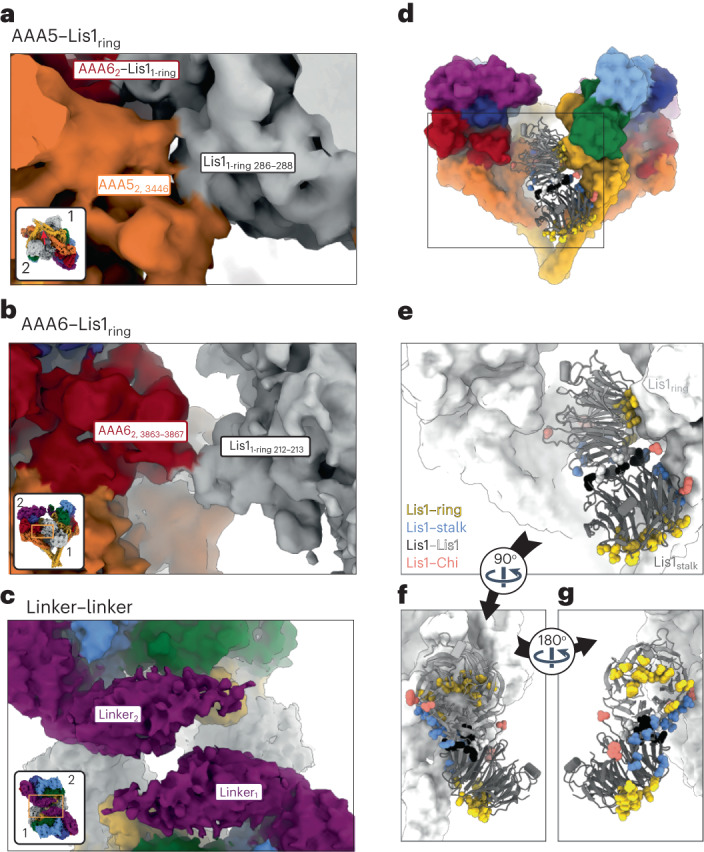
Interactions involved in stabilizing Chi. **a**–**c**, Chi is stabilized by three interfaces that, to the best of our knowledge, have not been previously reported: AAA5–Lis1_ring_ (a); AAA6–Lis1_ring_ (b); and a linker–linker interaction (c). In **a**, Chi is viewed from the dynein stalk and towards the motor domain. Figure 2b shows the location of these interactions in the context of full dynein. All three panels show the cryo-EM map colored by domain, with Lis1 in gray. The insets show the region of Chi that is highlighted in the main panel. **d**–**g**, Location of interaction interfaces in the Lis1_ring_–Lis1_stalk_ dimer. **d**, Our model of Chi. Dynein is shown in surface representation (filtered to 8 Å), and Lis1 is shown as a ribbon diagram. The area within the square is enlarged in **e**. **e**, Residues involved in the four Lis1 interactions (binding of Lis1 to site_ring_ and site_stalk_, the Lis1–Lis1 dimer interface, and the interactions stabilizing Chi) are shown as sphered and colored by interface. Dynein is shown in white. **f**, The Lis1 dimer is viewed facing the dynein monomer, with which the Lis1–ring and Lis1–stalk interactions are formed. The dynein in front was removed for clarity. **g**, The Lis1 dimer viewed from the other side, facing the dynein monomer with which the Chi-stabilizing interactions are made. The dynein in front was removed for clarity.

In Chi, the Lis1 proteins bound to the dynein motor domains act as wedges that keep the two dyneins apart, preventing most of the interactions that stabilize Phi (Fig. 2a,b and Extended Data Fig. 3b). The one exception is a contact between dynein’s stalk helices, which acts as a hinge where Phi opens to accommodate Lis1 and transition to Chi (Fig. 2a,b). The dynein–dynein Phi contacts that are disrupted in Chi are replaced by new contacts between Lis1 bound to site_ring_ on one dynein and the AAA5 (Fig. 3a) and AAA6 (Fig. 3b) domains of the opposite dynein. The AAA5 and AAA6 Chi-specific interactions involve residues on Lis1’s β-propeller that are different from those involved in all previously characterized Lis1–dynein and Lis1–Lis1 contacts (Fig. 3d–g and Supplementary Video 1). The Lis1 bound to site_stalk_ does not interact with the opposite dynein molecule in Chi (Fig. 1c,d).

**Extended Data Fig. 4:**
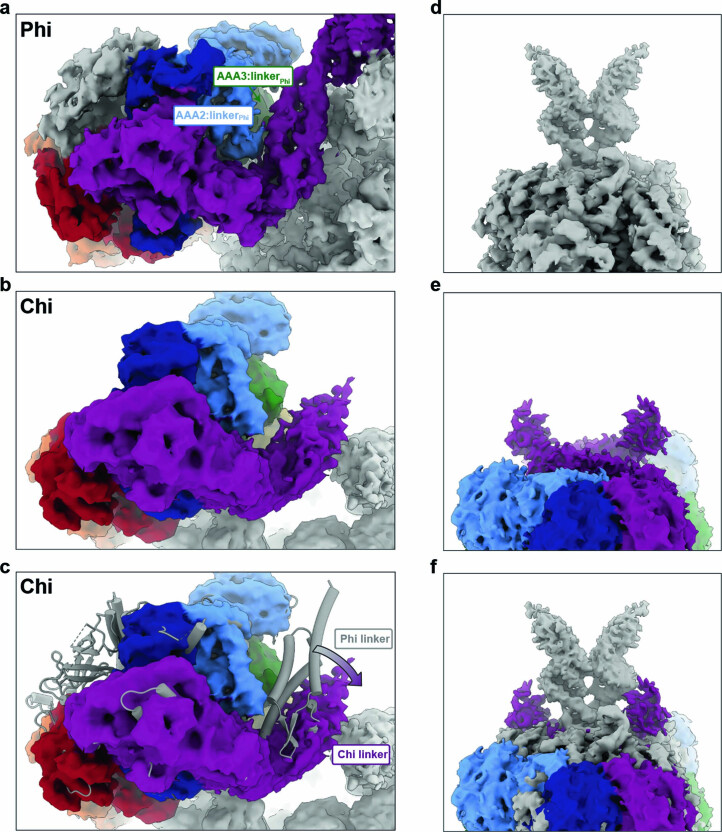
The conformation of the linker is different in Chi and Phi. **a**, The linker docks onto AAA2 and AAA3 in Phi. **b**, In contrast, the linker is disengaged from the AAA ring in Chi. **c**, Overlay of Phi (a) and Chi (b) highlights the different positions of the linker. **d**, **e**, The N-termini of the linker adopt different paths in Phi (d) and Chi (e). **f**, Overlay of Phi (d) and Chi (e) shows how the linkers are pulled apart in Chi.

The differences between Phi and Chi include changes in the conformation of the linker domain (Extended Data Fig. 4). In Phi dynein, the linker is bent and docked on AAA2 and AAA3 (Extended Data Fig. 4a), which is the same conformation seen in dynein alone in its ‘pre-power stroke’ state^6^. In contrast, in Chi, the linker is disengaged from AAA2 and AAA3 and shifted towards the opposing dynein molecule (Fig. 3c and Extended Data Fig. 4b,c); this accommodates the increased distance between the motor domains caused by the presence of Lis1 (Fig. 2c). This change in the linker conformation in turn causes a shift in the interface mediating the linker–linker interaction (Fig. 3c and Extended Data Fig. 4d–f); however, the resolution of the linker domain is too low to determine the exact nature of the residues involved.

The major features of the Chi structure—the disruption of most Phi-stabilizing interactions, a shift in the linker–linker interface, and the dependence of these on the presence of Lis1 and new interactions between Lis1 and dynein—suggest that Chi is an early intermediate in the activation of dynein that provides a mechanistic explanation for the role of Lis1 in this process. We set out to test this hypothesis and validate our structural model in vivo using *S. cerevisiae* and in vitro using recombinant human dynein–dynactin–activating adapter complexes.

### Dynein and Lis1 Chi contacts are required in vivo in yeast

**Fig. 4:**
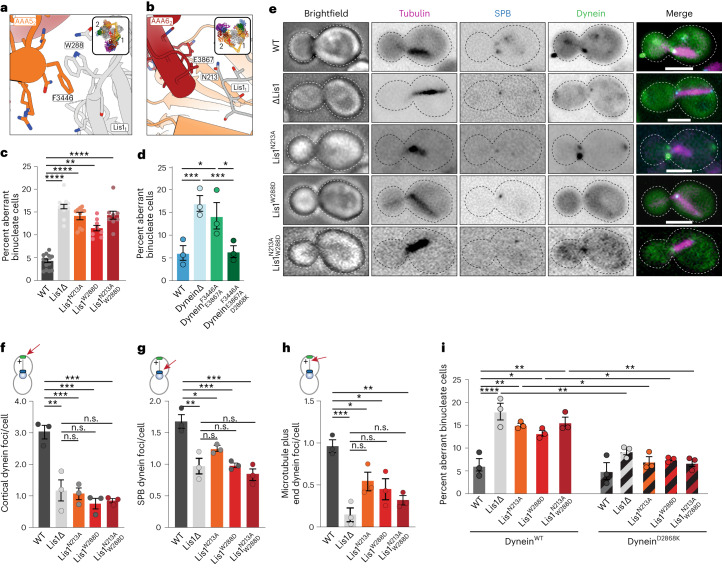
Role of Chi in dynein’s function in vivo in *S. cerevisiae*. **a**, The yeast Chi AAA5–Lis1_ring_ interface. Trp288 in Lis1 was mutated to Asp. **b**, The yeast Chi AAA6-Lis1_ring_ interface. Asn213 was mutated to Ala. We did not build the side chain of E3867 beyond its β-carbon owing to the lower resolution of this part of the map. **c**, Quantitation (mean ± s.e.m.) of the percentage of cells displaying an aberrant binucleate phenotype for wild type (WT, dark gray), Lis1 deletion (light gray), Lis1^N213A^ (orange), Lis1^W288D^ (red) or Lis1^N213A W288D^ (maroon). WT *n* = 12, ΔLis1 *n* = 11, Lis1^N213A^ *n* = 10, Lis1^W288D^, and Lis1^N213A, W288D^ *n* = 8 biological replicates from independent cultures, with at least 200 cells per condition in each replicate. Statistical analysis was done using a one-way ANOVA with Tukey’s multiple comparison test. WT and ΔLis1 ****P < 0.0001, WT and Lis1^N213A^ ****P < 0.0001, WT and Lis1^W288D^ **P = 0.0001, WT and Lis1^N213A W288D^ ****P < 0.0001. Differences not noted are not statistically significant. **d**, Quantitation (mean ± s.e.m.) of the percentage of cells displaying an aberrant binucleate phenotype for wild type (blue), Dyn1 deletion (light blue), Dyn1^F3446A, E3867A^ (green), and Dyn1^F3446A, E3867A, D2868K^ (dark green). *n* = 3 biological replicates, with at least 200 cells per condition per replicate. Statistical analysis was done using a one-way ANOVA with Tukey’s multiple comparison test. WT and ΔDyn1 ***P = 0.0002, WT and Dyn1^F3446A E3867A^ *P = 0.01, ΔDyn1 and Dyn1^F3446A E3867A D2868K^ ***P = 0.0002, Dyn1^F3446A E3867A^ and Dyn1^F3446A E3867A D2868K^ *P = 0.015. **e**, Example images of endogenous dynein localization (*DYN1-3XGFP*) in dividing yeast cells expressing a fluorescently tagged SPB (*SPC110-tdTomato*) component and tubulin (*TUB1-CFP*). Scale bars are 3 μm. **f**–**h**, Quantification of dynein localization. Graphs show the number (mean ± s.e.m.) of dynein foci per cell localized to the cortex (**f**), SPB (**g**), and microtubule plus ends (**h**) in WT, Lis1Δ, Lis1^N213A^, Lis1^W288D^, and Lis1^N213A W288D^ yeast strains, from three biological replicates. Statistical analysis was performed on the means of each biological replicate using a one-way ANOVA with Tukey’s multiple comparison test. **f**, WT and ΔLis1 **P = 0.0011, WT and Lis1^N213A^ ***P = 0.0007, WT and Lis1^W288D^ ***P = 0.0002, WT and Lis1^N213A W288D^ ***P = 0.003. **g**,WT and ΔLis1 **P = 0.0023, WT and Lis1^N213A^ *P = 0.0396, WT and Lis1^W288D^ ***P = 0.0009, WT and Lis1^N213A W288D^ ***P = 0.0003. **h**, WT and ΔLis1 ***P = 0.0006, WT and Lis1^N213A^ *P = 0.02, WT and Lis1^W288D^ *P = 0.049, WT and Lis1^N213A W288D^ ***P = 0.0039. n.s. (not significant), *P* = 0.069 to >0.9981. *n* ≥ 40 cells per biological replicate; >120 cells total per condition. **i**, Quantitation (mean ± s.e.m.) of the percentage of cells displaying an aberrant binucleate phenotype for wild type (WT, dark gray), Lis1 deletion (light gray), Lis1^N213A^ (orange), Lis1^W288D^ (red), Lis1^N213A W288D^ (maroon), Lis1 WT with the Phi-breaking dynein mutant dynein^D2868K^ (striped dark gray), Lis1 deletion with dynein^D2868K^ (striped light gray), Lis1^N213A^ with dynein^D2868K^ (striped orange), Lis1^W288D^ with dynein^D2868K^ (striped red), and Lis1^N213A W288D^ with dynein^D2868K^ (striped maroon). The WT condition is the same as in **d**. *n* = 3 biological replicates, with at least 200 cells per condition per replicate. Statistical analysis was done using a one-way ANOVA with Tukey’s multiple comparison test. WT and ΔLis1 ****P < 0.0001, WT and Lis1^N213A^ **P = 0.0035 WT and Lis1^W288D^ *P = 0.045, WT and Lis1^N213A W288D^ **P = 0.0014, ΔLis1 and ΔLis1 with dynein^D2868K^ **P = 0.0046, Lis1^N213A W288D^ and Lis1^N213A W288D^ with dynein^D2868K^ **P = 0.0036, Lis1^N213A^ and Lis1^N213A^ with dynein^D2868K^ *P = 0.0134, Lis1^W288D^ and Lis1^W288D^ with dynein^D2868K^ *P = 0.0244, Lis1^N213A, W288D^. Differences not noted are not statistically significant. Source data

**Extended Data Fig. 5:**
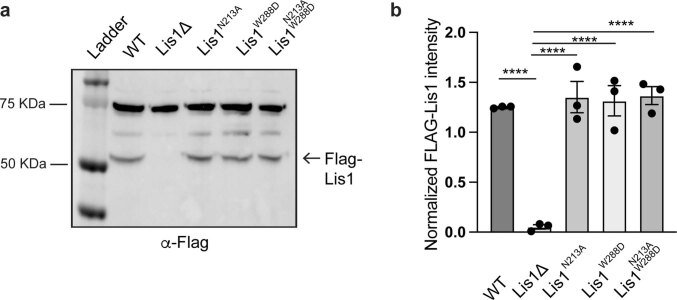
Western blot analysis of Lis1 mutant expression. **a**, Cell lysates from wild type (WT), Lis1Δ or mutant Lis1 (Lis1^N213A^, Lis1^W288D^ and Lis1^N213A, W88D^) *S. cerevisiae* strains containing Flag-tagged Lis1 (Pac1) were immunoprecipitated using anti-Flag agarose beads and immunoblotted for the FLAG peptide (α-FLAG). **b**, Quantitative analysis (mean ± s.e.m.) of Lis1 expression (α-FLAG band) normalized against the non-specific band observed at ~75 KDa for three independent immunoprecipitations. Statistics were generated using a One-way ANOVA with Tukey multiple comparisons of each mean test. **** WT and ΔLis1, ΔLis1 and Lis1^N213A^, ΔLis1 and Lis1^W288D^, ΔLis1 and Lis1^N213A, W288D^ p < 0.0001. Differences not noted are not statistically significant. Source data are provided with the manuscript. Source data

We first sought to determine whether the new contact sites between Lis1 and dynein at AAA5 (Fig. 4a) and AAA6 (Fig. 4b) that form Chi are important for the dynein pathway in vivo. For this, we turned to experiments in *S. cerevisiae*. In yeast, dynein functions to align the mitotic spindle, such that, upon the completion of mitosis, both the mother and daughter cell inherit a nucleus^18,20,41–43^. Deletion of the gene encoding dynein (*DYN1*; GeneID: 853928) and dynactin subunits, as well as Lis1, causes nuclear segregation defects that are readily quantifiable because they produce binucleate cells^22^. To disrupt the Chi-specific interactions (Fig. 4a,b), we mutated Asn213 in Lis1 to Ala (Lis1^N213A^), Trp288 to Asp (Lis1^W288D^), or both (Lis1^N213A W288D^) in the endogenous locus of yeast Lis1 (*PAC1*; GeneID: 854443). Western blots of strains containing FLAG-tagged versions of the mutants confirmed that Lis1^N213A^, Lis1^W288D^, and Lis1^N213A W288D^ were expressed at wild-type levels (Extended Data Fig. 5a,b). We found that, among cells expressing any of these three mutants, the percentage of cells containing two nuclei was increased, similar to cells in which Lis1 was deleted (Fig. 4c). We obtained similar results when we disrupted Chi on dynein’s side. On the basis of our structure, we introduced F3446A in AAA5 and E3867A in AAA6 (Dyn1^F3446A E3867A^; Fig. 4a,b). Although we did not build the side chain for E3867 in our model owing to the lower resolution of that part of the map, this residue is a good candidate for mediating the interaction between dynein and Lis1-N213. These substitutions in dynein resulted in an increase in the percentage of binucleate cells similar to that observed following deletion of dynein (Fig. 4d). This suggests that the dynein–Lis1 interactions involved in forming Chi are required for Lis1’s regulation of dynein in vivo.

### Dynein localization in yeast depends on dynein and Lis1 Chi contacts

Next, we wanted to determine how the Lis1–dynein interactions involved in forming Chi contribute to dynein’s localization in yeast cells (Fig. 4e–h). Yeast dynein assembles into fully active complexes with dynactin and Num1, the presumed yeast dynein activating adapter, at the cell cortex^18,44^. From this site, active dynein pulls on spindle pole body (SPB)-attached microtubules to align the mitotic spindle, which ultimately favors equal segregation of nuclei between mother and daughter cells. Dynein reaches the cortex by first localizing to microtubule plus ends, through either kinesin-dependent transport or cytosolic recruitment^45–47^. Dynein also localizes to the SPB, where microtubule minus ends are found^46^. Lis1 is required for dynein’s localization to all three sites^20,22,48^. To assess how the mutants that target the new interactions between dynein and Lis1 in Chi affect dynein localization, we introduced Lis1^N213A^, Lis1^W288D^ and Lis1^N213A W288D^ at the endogenous Lis1 (*PAC1*) locus. In these strains, dynein, α-tubulin, and a spindle pole body component were also tagged with a fluorescent protein (*DYN1-3XGFP*, *TUB1-CFP*, and *SPC110-tdTomato*, respectively) (Fig. 4e)^22,30^ and used to measure the number of dynein foci that were found at the cell cortex (Fig. 4f), SPBs (Fig. 4g), or microtubule plus ends (Fig. 4h) in each cell. All three mutants showed a striking reduction in the number of dynein foci at the cell cortex, similar to the strain with Lis1 deletion (Fig. 4f). Because the cortex is the site of dynein–dynactin–Num1 assembly, these data suggest that the Chi conformation is important for the assembly of active dynein complexes in vivo. All three mutants also significantly affected the ability of dynein to localize to microtubule plus ends. One mechanism dynein uses to reach microtubule plus ends is through kinesin transport in a complex that also contains Lis1 and Bik1/CLIP170 (refs. ^20,49,50^). Thus, it is possible that this transport complex uses the dynein–Lis1 interactions we identified in Chi, or potentially the Chi conformation itself. Together, our data show that the contacts we observe between dynein and Lis1 in the Chi structure are required for active dynein complex assembly and function in vivo.

### Phi-disrupting mutations bypass the need for Chi in yeast

We hypothesize that Chi follows Phi as an intermediate in dynein’s activation pathway. This model predicts that mutations that prevent dynein from forming the autoinhibited Phi conformation should bypass the need for Chi to form. We tested this using genetic epistasis experiments. We generated yeast double mutant strains containing each of our Chi-breaking Lis1 substitutions (Lis1^N213A^, Lis1^W288D^, or Lis1^N213A,W288D^) combined with a Phi-breaking substitution in the endogenous dynein/*DYN1* locus (dynein^D2868K^)^21^, and performed nuclear segregation assays. In agreement with our model, introducing dynein with the Phi-breaking substitution into the strains carrying the Chi-breaking Lis1 mutants restored the percentage of cells with two nuclei to wild-type levels (Fig. 4i). We observed the same when dynein^D2868K^ was introduced into the Lis1-deletion strain (Fig. 4i). Introducing the Phi-breaking substitution into dynein already carrying Chi-disrupting alterations (dynein^F3446A E3867A D2868K^) also fully rescued the increase in the percentage of binucleate cells (Fig. 4d).

### Dynein and LIS1 Chi contacts are required for human dynein complex assembly

**Fig. 5:**
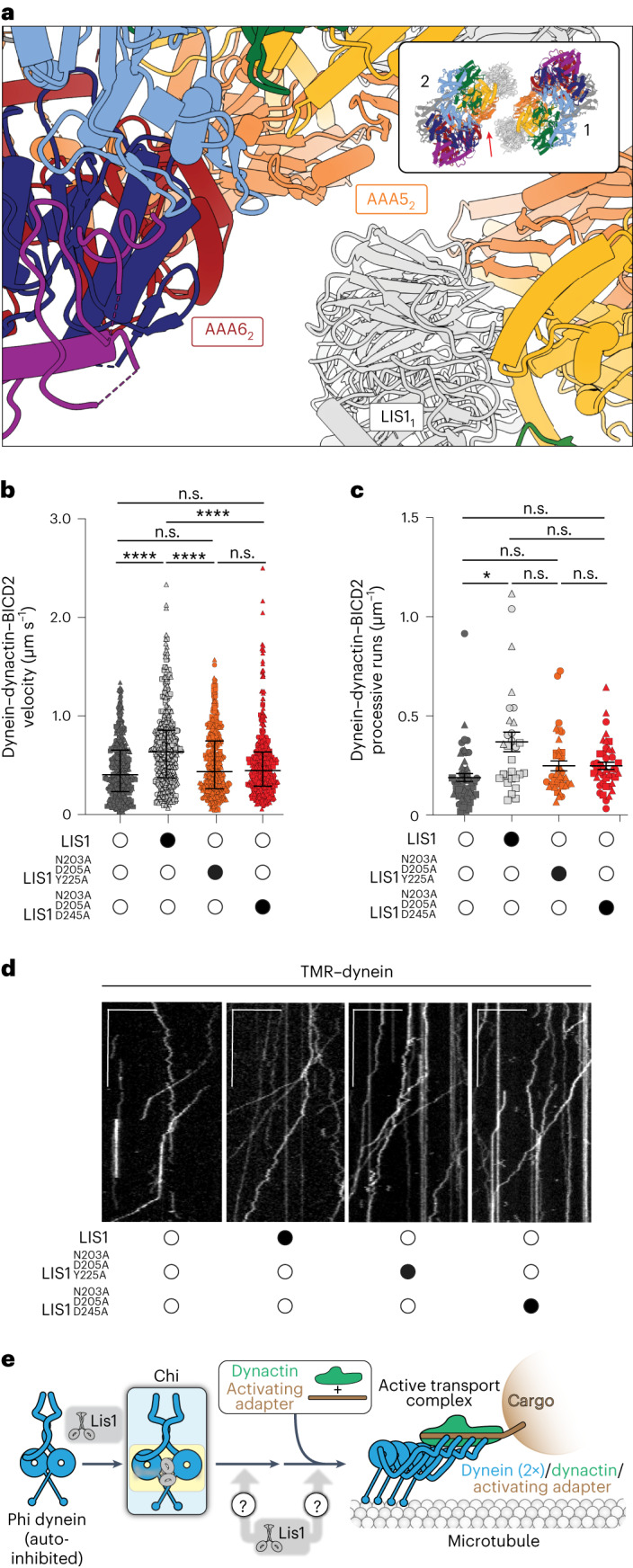
Role of Chi in human dynein’s ability to move on microtubules. **a**, A model of the human Chi AAA6_2_ and AAA5_2_–LIS1_1_ interface. The arrow in the inset indicates the area highlighted in the main panel. **b**, Single-molecule velocity (median ± interquartile range) of TMR–dynein–dynactin–BICD2 complexes in the absence (white circles) or presence (black circles) of different human LIS1 constructs. The data points are represented as triangles, circles, squares, and hexagons corresponding to single measurements within each technical replicate. Four technical replicates were collected for each condition, and the number of data points (*n*) per each replicate is listed (no LIS1, *n* = 142, 91, 152, 131; LIS1, *n* = 147, 118, 119, 120; LIS1^N203A D205A Y225A^, *n* = 149, 91, 120, 103; LIS1^N203A D205A D245A^, *n* = 147 109 128 108). No LIS1 and LIS1 ***P = 0.0002, LIS1 and LIS1^N203A D205A Y225A^ **P = 0.0051, LIS1 and LIS1^N203A D205A D245A^ **P = 0.004. One-Way ANOVA with Tukey’s multiple comparison test. **c**, Processive runs (mean ± s.e.m.) of TMR–dynein–dynactin–BICD2 complexes in the absence (white circle) or presence (black circle) of different unlabeled human LIS1 constructs. The data points are represented as triangles, circles, squares, and hexagons corresponding to single measurements within each technical replicate. Four replicates per condition were collected, and the number of data points (*n*) per each replicate is listed (no LIS1, *n* = 14, 20, 20, 8; LIS1, *n* = 7, 16, 7, 5; Lis1^N203A D205A Y225A^, *n* = 15, 7, 16, 9; Lis1^N203A, D205A D245A^, *n* = 17, 9, 17, 9). No LIS1 and LIS1 *P = 0.023. One-Way ANOVA with Tukey’s multiple comparison test. **d**, Representative kymographs from single-molecule motility assays with purified TMR–dynein–dynactin–BICD2 in the absence (white circle) or presence (black circle) of different human LIS1 constructs. Scale bars, 10 μm (*x*) and 40 s (*y*). **e**, Schematic of the hypothesis for how Lis1 relieves dynein autoinhibition. Source data

**Extended Data Fig. 6:**
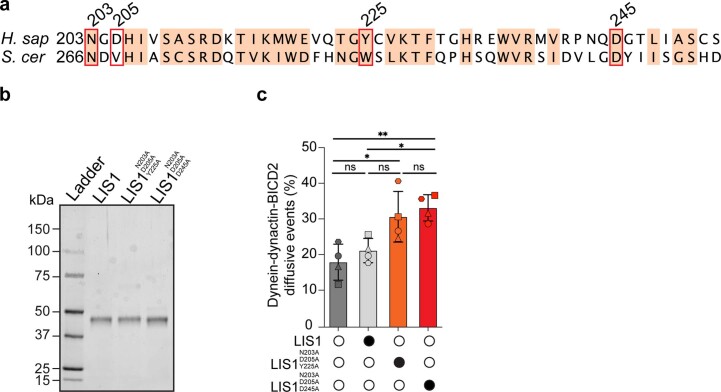
Chi mutations in human LIS1. **a**, Sequence conservation between *S. cerevisiae* (*S. cer*) and human (*H. sap*) Lis1 in the vicinity of the dynein binding site in the Chi structure. Residues with 70% conservation or higher are shaded in light red. The residues mutated in this study: N203, D205, Y225, and D245 are highlighted by red boxes. **b**, Sodium dodecyl sulfate–polyacrylamide gel electrophoresis (SDS–PAGE) of the human LIS1 constructs used for motility assays. **c**, Comparison of percent diffusive events (mean ± SEM) from single-molecule TMR-dynein-dynactin-BICD2 assays performed in the absence (white circle) or presence (black circle) of different LIS1 constructs. The data points are represented as triangles, circles, and squares, corresponding to the mean diffusive events measured within each technical replicate. Four replicates per condition were collected and the number of data points (n) per each replicate is listed (no LIS1, n = 14/20/20/8; LIS1, n = 7/16/7/5; LIS1^N203A, D205A, Y225A^, n = 15/7/16/9; LIS1^N203A, D205A, D245A^, n = 17/9/17/9). ** no LIS1 and LIS1^N203A, D205A, D245A^ p = 0.0051, * no LIS1 and LIS1^N203A, D205A, Y225A^ p = 0.017, * LIS1 and LIS1^N203A, D205A, D245A^ ** p = 0.025 ns, not statistically significant, One-Way ANOVA with Tukey multiple comparisons of each mean. Source data are provided with the manuscript. Source data

To assess the importance of the Chi conformation, or more generally of the new dynein–Lis1 interaction involved in stabilizing it, in the assembly of active human dynein–dynactin–activating adapter complexes at the molecular level, we next turned to reconstituting the complexes using human proteins. In vitro, human Lis1 protein (herein, LIS1) enhances the formation of dynein–dynactin–activating adapter complexes containing two dynein dimers, which move faster than the single dynein dimer complexes that form in the absence of LIS1 (refs. ^14,15^). To do this, we first had to identify Chi-disrupting substitutions in human LIS1 that were predicted to be equivalent to the ones we identified in yeast. Directly overlaying our recent structure of a human dynein–LIS1 complex^51^ onto each motor domain of yeast Chi did not work, as LIS1 bound to site_ring_ is too far away from the opposite dynein to form the Chi-stabilizing interactions with AAA5 and AAA6 (Fig. 5a). This difference is due to human LIS1 having shorter peripheral loops and being slightly rotated relative to yeast Lis1 (ref. ^51^) when bound to dynein. To circumvent this problem, we modeled human Chi by aligning a copy of our structure of human dynein bound to two LIS1s to each motor domain in Chi dynein, and then manually moving the models so that they were within interacting distance while still maintaining the stalk–stalk interaction observed in both Chi and Phi dynein. Using this model, we made two sets of human LIS1 substitutions: p.N203A, p.D205A, and p.Y225A to disrupt both the LIS1–AAA5 and LIS1–AAA6 interfaces, and p.N203A, p.D205A, and p.D245A to disrupt the LIS1–AAA6 interface alone. Two of the sites we mutated in human LIS1, N203 and D245, are also conserved in yeast; Y225 corresponds to the W288 that we mutated in yeast Lis1 (Extended Data Fig. 6a).

Next, we purified these mutant human LIS1 constructs (Extended Data Fig. 6b) and examined their ability to activate human dynein–dynactin complexes containing the BICD2 activating adapter in single-molecule motility assays. In these assays, activation of motility is read out by observing an increase in dynein complex velocity in the presence of LIS1, which results from the enhanced formation of dynein complexes containing two dynein dimers^13–15^. As we have shown previously, pre-incubation of dynein–dynactin–BICD2 with 300 nM wild-type human LIS1 increased the velocity and number of processive runs of these complexes (Fig. 5b–d)^15^. By contrast, there was no significant difference between dynein velocity and the number of processive runs when dynein–dynactin–BICD2 complexes were pre-incubated with the two human LIS1 mutants (LIS1^N203A D205A Y225A^ and LIS1^N203A D205A D245A^), as compared with complexes that were formed in the absence of LIS1 (Fig. 5b–d). We also observed an increase in the number of diffusive events in the presence of LIS1 mutants (Extended Data Fig. 6c). These data indicate that the dynein–Lis1 contact sites found in the Chi structure are important for human LIS1’s role in forming the activated human dynein–dynactin–activating adapter complex, either by relieving the autoinhibited Phi conformation or by playing a role in a later step in the assembly of activated complexes, or a combination of both.

## Discussion

On the basis of the data presented here, we propose that Chi is an intermediate state in the dynein activation pathway, providing the first structural and mechanistic explanation for how Lis1 relieves dynein autoinhibition. We propose that the wedging of Lis1 between the two dynein motor domains primes dynein for binding dynactin and activating adapter protein(s).

The Chi conformation has important implications for dynein activation. In dynein’s autoinhibited Phi state, the ‘tails,’ which precede the motor domain, make many contacts with each other until they reach their ‘neck’ region, where they move away from each other before rejoining again at the linker domain. Switching to the active dynactin-bound form requires the tail to undergo a large conformational change that turns the loose twofold symmetry present in Phi into the translational (‘parallel’) symmetry dynein adopts when bound to dynein and an activating adapter (Fig. 5e). This rearrangement involves breaking the linker–linker contacts, as well as many of the interactions between the tails and associated chains. We hypothesize that the Chi conformation primes dynein for binding dynactin and an activating adapter protein by stabilizing an intermediate conformation of the tail. In Chi, binding of Lis1 forces the linkers apart, which would cause some of these interactions to break. This would pull the dynein heavy chains apart from each other, disrupting the neck and potentially transmitting conformational changes all the way up the tail.

## Methods

### Electron microscopy sample preparation

Grids were prepared and imaged as previously described^31^. Briefly, monomeric yeast dynein^E2488Q^ was randomly biotinylated using water-soluble Sulfo ChromaLink biotin and then dialyzed into TEV buffer. Grid samples containing 150 nM dynein^E2488Q^, 650 nM Lis1, 1.2 mM ATP, and 1.2 mM Na_3_VO_4_ were applied to streptavidin affinity grids and vitrified^52,53^.

For the imaging of wild-type yeast dynein monomer in the presence of ATP and Lis1, purified fresh dynein (not frozen) was incubated with apyrase for 30 min (apyrase, 0.1 U mL^–1^, NEB) prior to gel filtration on a Superose 6 Increase column pre-equilibrated in buffer containing 50 mM Tris, pH 8, 150 mM KCl, 2 mM EDTA, and 1 mM DTT. Eluted protein was concentrated to about 5 µM and diluted 1:1 with Lis1 or TEV buffer and incubated on ice for 10 min (final concentrations: 2.5 µM dynein, 2.5 µM Lis1). After an additional 30-s incubation on ice in the presence of ATP (final concentration: 1.25 mM ATP) samples were applied to plasma-cleaned (Solarus, Gatan) UltrAuFoil Holey Gold R 1.2/1. µM3 grids (Quantifoil). A vitrobot (FEI) was used to blot excess sample and plunge freeze the grids in liquid ethane. Grids were stored in liquid nitrogen until they were ready to be imaged.

### Electron microscopy image collection and processing

Details on image collection and initial processing have been reported elsewhere^31^. Following particle picking in crYOLO5 (ref. ^54^), particles were extracted and binned to 3.93 Å pixel^–1^ in Relion 3.0 (ref. ^55^). Multiple rounds of 2D classification were carried out first in Relion 3.0 and subsequently in cryoSPARC^56^ to remove bad particles. Some class averages showed particles with more than one dynein molecule. Using particles from all good 2D classes, initial ab initio reconstructions failed to reconstruct a volume containing more than one dynein molecule. Particles belonging to 2D class averages that potentially contained more than one dynein molecule were manually selected and used in an ab initio reconstruction that resulted in an initial map for Chi dynein bound to Lis1. This volume was used together with our previously published map of monomeric dynein^E2488Q^ bound to Lis1 (EMDB-23829) to separate monomeric dynein^E2488Q^ from Chi dynein using heterogeneous refinement. We performed one additional round of heterogeneous refinement to further remove any monomeric dynein molecules. Using our final particles, we ran non-uniform refinement with *C*_2_ symmetry and optimized per-group CTF parameters enabled to give us a 4.1-Å map.

Wild-type yeast dynein monomer incubated in the presence of ATP and Lis1 was imaged using a Talos Arctica operated at 200 kV and equipped with a K2 Summit direct electron detector (Gatan). Automated data collection was performed using Leginon. A total of 4,525 movies was collected at ×36,000 magnification (1.16 Å pixel^–1^). The dose was ~4.63 e^–^ Å^–2^ s^–^^1^ with a total exposure time of 11 s divided into 200 ms frames, for a total of 40 frames. The defocus range was set to 0.8–2.4 µm. All movies were aligned in CryoSPARC live^57^ using MotionCor2 (ref. ^58^) of the dose-weighted frames. CTF was estimated in dose-weighted images using CTFFIND4 (ref. ^59^). Images with CTF fits worse than 5 Å were excluded from further processing. Particles were initially selected using blob finder in CryoSPARC, and these peaks were used for Topaz^60^ model training and final particle picking using Topaz. Particles were extracted and binned to 4.64 Å pixel^–1^. Multiple rounds of 2D classification, with a varying number of online-EM interactions (30–40) and a batch size per class of between 200 and 400 dependent on the number of particles in each classification were carried out in cryoSPARC to remove bad particles.

### Model building and refinement

Symmetry expansion followed by local refinement was used to improve the overall resolution of the motor domain to 3.6 Å. The yeast dynein^E2448Q^–(Lis1)_2_ model (PDB 7MGM) was docked, and rigid body fit into the map using Phenix real space refine^61^. Discrepancies between the model and map were fixed manually in COOT6 (ref. ^62^) and then refined using a combination of Phenix real space refine and Rosetta Relax (v.13)^63^. Once the monomer model was finished, it was placed in the original map and refined using a similar strategy.

The resolution of the Chi map did not allow us to distinguish between ATP and ADP.Vi. Therefore, we chose to model ATP at AAA1, AAA2, and AAA3, to be consistent with our previously published structure of the yeast dynein motor domain bound to two Lis1s, which was obtained from the same data set^31^.

### Cloning, plasmid construction, and mutagenesis

The pDyn1 plasmid (pACEBac1 expression vector containing insect cell codon-optimized dynein heavy chain (*DYNC1H1*) fused to a His-ZZ-TEV tag on the amino terminus and a carboxy terminal SNAPf tag (New England Biolabs) and the pDyn2 plasmid (the pIDC expression vector with codon-optimized *DYNC1I2*, *DYNC1LI2*, *DYNLT1*, *DYNLL1*, and *DYNLRB1*) were a gift from A. Carter (LMB-MRC). The pDyn1and pDyn2 plasmids were recombined in vitro with a Cre recombinase (New England Biolabs) to generate the pDyn3 plasmid. The presence of all six dynein chains was verified by PCR. The pFastBac plasmid with codon-optimized human full-length Lis1 (*PAFAH1B1*) fused to an N-terminal His-ZZ-TEV tag was a gift from A. Carter (LMB-MRC). The BICD2s construct (amino acids 25–398) fused to sfGFP on the N terminus and inserted into a pET28a expression vector was obtained as described previously^17^. Substitutions in LIS1 were made using a multi-site-directed mutagenesis kit (Agilent) and the following primers: 5′-TGTGAATGTCTTCACACAGGCGCCAGTTTGCACTTCCCAC-3′, 5′-GGTACGGCCAAATCAAGCTGGCACTCTGATAGCCAG-3′, and 5′-CAGTAGCCATCATGCCCGCTGGAGCTCATATAGTGTCTGCCTC-3′. The yeast gene encoding Lis1 (*PAC1*) previously cloned into a Topo 2.1 vector using the TOPO TA Cloning Kit (Thermo Fisher), was mutated using a site-directed mutagenesis kit (Agilent) with the primers: 5′-GGATGTTTTATTTACCAATTATACGGCCTCCAGCAAGAAGAACTATTTGGTG-3′ and 5′-cACCAAATAGTTCTTCTTGCTGGAGGCCGTATAATTGGTAAATAAAACATCC-3′ to insert the N213A substitution. *PAC1* or *pac11-N213A* were mutated using the primers 5′-TTTGGGACTTCCACAATGGTGACTCGTTGAAAACATTTCAGCC-3′ and 5′-GGCTGAAATGTTTTCAACGAGTCACCATTGTGGAAGTCCCAAA-3′ to insert the W288D substitution. *DYN1(3396–3921aa)* was cloned into a pBlueHeron vector through Gibson assembly and was mutagenized with the primers 5′-GATTAGAAAATGCAATTAGAGCCGGAAGTGTAGTTATAATTC-3′ and 5′-GAATTATAACTACACTTCCGGCTCTAATTGCATTTTCTAATC-3′ to introduce the F3446A substitution. *dyn1-F3446A* was mutated using a site-directed mutagenesis kit (Agilent) with the primers 5′-GAGGAGACAAAGGCGGCAGAAGCACATGAGAAATTCAAAATGT-3′ and 5′-ACATTTTGAATTTCTCATGTGCTTCTGCCGCCTTTGTCTCCTC-3′ to introduce the E3867A substitution.

### Yeast strains

The *S. cerevisiae* strains used in this study are listed in Supplementary Table 1. The endogenous genomic copy of *PAC1* (encoding Lis1) was deleted using PCR-based methods as previously described^64^. In brief, *K. lactis URA3* with homology arms complementary to regions upstream and downstream of the *PAC1* or *DYN1* genomic locus was generated using PCR. This fragment was transformed into a strain with the preferred genetic background using the lithium acetate method^65^ and screened by colony PCR. Point mutants were generated using QuikChange site-directed mutagenesis (Agilent) and verified by DNA sequencing. Mutated fragments were re-inserted into the kl*URA3* strains to reintroduce the mutated *PAC1* or *DYN1* gene. Positive clones lacking URA3 (kl*URA3*-) were selected in the presence of 5-fluoroorotic acid, screened by colony PCR, and verified by DNA sequencing.

### Nuclear segregation assay

Single colonies were picked and grown at 30 °C. Log-phase *S. cerevisiae* cells growing at 30 °C were transferred to 16 °C for 16 h. Cells were fixed with 75% ethanol for 1 h, sonicated for 5 s at 40% amplitude, and mounted in medium containing DAPI. Imaging was performed using an Apo TIRF 100 ×1.49 NA objective (Nikon, Plano Apo) on a Nikon Ti2 microscope with a Yokogawa-X1 spinning disk confocal system, MLC400B laser engine (Agilent), Prime 95B back-thinned sCMOS camera (Teledyne Photometrics), and a piezo Z-stage (Mad City Labs). The samples were blinded during imaging. The percentage of aberrant binucleate cells was calculated as the number of binucleate cells divided by the sum of wild-type and binucleate cells. Eight to 12 biological replicates from independent colonies were done for Figure 4c, and three were done for Figure 4d,i. At least 200 cells were counted for each biological replicate per condition.

### Live-cell imaging

Single colonies were picked and grown at 30 °C. Log-phase live *S. cerevisiae* cells were mounted on a thin agarose pad made from SC medium pressed between two glass slides. Live cells genetically modified to express fluorescently labeled DYN1-3XGFP, CFP-TUB1, and SPC110-tdTomato were imaged using a Yokogawa W1 confocal scanhead mounted to a Nikon Ti2 microscope with an Apo TIRF 100 ×1.49 NA objective (Nikon, Plano Apo). The microscope was run with NIS Elements using the 488 nm 515 nm and 561 nm lines of a six-line (405 nm, 445 nm, 488 nm, 515 nm, 561 nm, and 640 nm) LUN-F-XL laser engine and Prime95B cameras (Photometrics). The DYN1-3×GFP foci localizing to the spindle pole body (SPB), microtubule plus end, and cell cortex were outlined as regions of interest in Fiji^66^, recorded, and analyzed for three replicates of at least 120 cells for each sample.

### *S. cerevisiae* immunoprecipitations and western blots

Log-phase *S. cerevisiae* cells grown at 30 °C were pelleted at 4,000*g*, resuspended in water, and flash frozen in liquid nitrogen. Liquid-nitrogen-frozen yeast cell pellets were lysed by grinding in a chilled coffee grinder, resuspended in dynein-lysis buffer (30 mM HEPES pH 7.4, 50 mM potassium acetate, 2 mM magnesium acetate, 1 mM EGTA, 10% glycerol, 1 mM DTT) supplemented with 1 mM Pefabloc, 0.2% Triton X-100, cOmplete EDTA-free protease inhibitor cocktail tablet (Roche), and 1 mM Pepstatin A (Cayman Chemical Company), and spun at 50,000*g* for 1 h. The protein concentration of the clarified supernatants was quantified using a Bradford Protein Assay (Bio-Rad), and equal amounts of clarified lysates were incubated with anti-FLAG M2 Affinity Gel (Sigma) overnight at 4 °C. Beads were washed with dynein-lysis buffer, boiled in SDS sample buffer, and loaded onto a NuPAGE Bis-Tris gel (Invitrogen). Gels were transferred to a PVDF membrane that was blocked with PBS-T (PBS1X and 0.1% Tween-20) containing 5% milk and 1% BSA for 1 h at room temperature and blotted with a rabbit anti-FLAG antibody (1:3,000; Proteintech 20543-1-AP) overnight at 4 °C. Membranes were then incubated with a goat-anti-rabbit IRDye 6800RD secondary antibody (LI-COR) and were scanned in a ChemiDoc Imaging system (Bio-Rad).

### Protein expression and purification

#### *S. cerevisiae* dynein

Protein purification steps were done at 4 °C unless otherwise indicated. *S. cerevisiae* dynein constructs were purified from *S. cerevisiae* using a ZZ tag as previously described^67^. Briefly, liquid-nitrogen-frozen yeast cell pellets were lysed by grinding in a chilled coffee grinder and resuspended in dynein-lysis buffer supplemented with 0.1 mM Mg-ATP, 0.5 mM Pefabloc, 0.05% Triton X-100, and cOmplete EDTA-free protease inhibitor cocktail tablet (Roche). The lysate was clarified by centrifuging at 264,900*g* for 1 h. The clarified supernatant was incubated with IgG Sepharose beads (GE Healthcare Life Sciences) for 1 h. The beads were transferred to a gravity flow column, washed with dynein-lysis buffer supplemented with 250 mM potassium chloride, 0.1 mM Mg-ATP, 0.5 mM Pefabloc, and 0.1% Triton X-100, and with TEV buffer (10 mM Tris-HCl pH 8.0, 150 mM potassium chloride, 10% glycerol, 1 mM DTT, and 0.1 mM Mg-ATP). Dynein was cleaved from IgG beads via incubation with 0.15 mg mL^–1^ TEV protease (purified in the Reck-Peterson lab) overnight at 4 °C. Cleaved dynein was concentrated using 100 kDa MWCO concentrator (EMD Millipore), filtered by centrifugation with Ultrafree-MC VV filter (EMD Millipore) in a tabletop centrifuge, and flash frozen in liquid nitrogen.

#### *S. cerevisiae* Lis1

*S. cerevisiae* Lis1 was purified from *S. cerevisiae* using 8×His and ZZ tags as previously described^32^. In brief, liquid-nitrogen-frozen pellets were ground in a pre-chilled coffee grinder, resuspended in buffer A (50 mM potassium phosphate pH 8.0, 150 mM potassium acetate, 150 mM sodium chloride, 2 mM magnesium acetate, 5 mM β-mercaptoethanol, 10% glycerol, 0.2% Triton X-100, 0.5 mM Pefabloc) supplemented with 10 mM imidazole (pH 8.0) and cOmplete EDTA-free protease inhibitor cocktail tablet, and spun at 118,300*g* for 1 h. The clarified supernatant was incubated with Ni-NTA agarose (QIAGEN) for 1 h. The Ni beads were transferred to a gravity column, washed with buffer A + 20 mM imidazole (pH 8.0), and eluted with buffer A + 250 mM imidazole (pH 8.0). The eluted protein was incubated with IgG Sepharose beads for 1 h. IgG beads were transferred to a gravity flow column and washed with buffer A + 20 mM imidazole (pH 8.0) and with modified TEV buffer (50 mM Tris-HCl pH 8.0, 150 mM potassium acetate, 2 mM magnesium acetate, 1 mM EGTA, 10% glycerol, 1 mM DTT). Lis1 was cleaved from the IgG beads by the addition of 0.15 mg mL^–1^ TEV protease (purified in the Reck-Peterson lab) for 1 h at 16 °C. Cleaved proteins were filtered by centrifuging with Ultrafree-MC VV filter (EMD Millipore) in a tabletop centrifuge and flash frozen in liquid nitrogen.

#### Human dynein

Full-length human SNAP-tagged dynein was expressed in Sf9 cells, as described previously^11,15^. Briefly, frozen Sf9 cell pellets from 2× 600 mL culture were resuspended in 80 mL of dynein-lysis buffer with 0.1 mM Mg-ATP, 0.5 mM Pefabloc, 0.05% Triton X-100, and cOmplete EDTA-free protease inhibitor cocktail tablet (Roche) and lysed using a Dounce homogenizer (10 strokes with a loose plunger and 15 strokes with a tight plunger). The lysate was clarified by centrifuging at 183,960*g* for 88 min in a Type 70 Ti rotor (Beckman). The clarified supernatant was incubated with 4 mL of IgG Sepharose 6 Fast Flow beads (GE Healthcare Life Sciences) for 3–4 h on a roller. The beads were transferred to a gravity flow column, washed with 200 mL of dynein-lysis buffer and 300 mL of TEV buffer (50 mM Tris-HCl pH 8.0, 250 mM potassium acetate, 2 mM magnesium acetate, 1 mM EGTA, 1 mM DTT, 0.1 mM Mg-ATP, 10% (vol/vol) glycerol). For fluorescent labeling of the C-terminal SNAPf tag, dynein-coated beads were labeled with 5 µM SNAP-Cell-TMR (New England Biolabs) in the column for 10 min at room temperature, and unbound dye was removed with a 300 mL wash with TEV buffer at 4 °C. The beads were then resuspended and incubated in 15 mL of TEV buffer supplemented with 0.5 mM Pefabloc and 0.2 mg mL^–1^ TEV protease (purified in the Reck-Peterson lab) overnight on a roller. The supernatant containing cleaved protein was concentrated using a 100 kDa MWCO concentrator (EMD Millipore) to 500 µL and purified via size-exclusion chromatography on a TSKgel G4000SWXL column (TOSOH Bioscience) with GF150 buffer (25 mM HEPES pH 7.4, 150 mM potassium chloride, 1 mM magnesium chloride, 5 mM DTT, 0.1 mM Mg-ATP) at 1 mL min^–1^. The peak fractions were collected, buffer-exchanged into a GF150 buffer supplemented with 10% glycerol, concentrated to 0.1–0.5 mg mL^–1^ using a 100 kDa MWCO concentrator (EMD Millipore). Purity was evaluated on SDS–PAGE gels, and protein aliquots were snap frozen in liquid N_2_ and stored at –80 °C.

#### Human dynactin

Dynactin (p62-HaloTag-3×FLAG) was purified from a stable 293T cell line, as previously described^11,15,17^. Briefly, frozen pellets from 293T cells (160 × 15-cm plates) were resuspended in dynein-lysis buffer supplemented with 0.1 mM Mg-ATP, 0.5 mM Pefabloc, 0.05% Triton, and cOmplete EDTA-free protease inhibitor cocktail tablet (Roche), and gently mixed at 4 °C for 15 min. The lysed cells were then centrifuged at 500,000*g* in a Ti70 rotor (Beckman) at 4 °C for 30 min. The clarified lysate was retrieved and added to 3 mL packed anti-FLAG M2 agarose resin (Sigma) and incubated with gentle mixing at 4 °C for 16 h. After incubation, the lysate-resin mixture was centrifuged at 1,000*g* for 2 min at 4 °C to pellet the resin, and the supernatant was decanted. The resin was transferred to a column at 4 °C, and the column was washed with 100 mL low-salt wash buffer (30 mM HEPES, pH 7.4; 50 mM potassium acetate; 2 mM magnesium acetate; 1 mM EGTA, pH 7.5; 10% glycerol; 1 mM DTT; 0.5 mM ATP; 0.5 mM Pefabloc; 0.02% Triton X-100), 100 mL high-salt wash buffer (30 mM HEPES, pH 7.4; 250 mM potassium acetate; 2 mM magnesium acetate; 1 mM EGTA, pH 7.5; 10% glycerol; 1 mM DTT; 0.5 mM ATP; 0.5 mM Pefabloc; 0.02% Triton X-100), and finally with 50 mL of low-salt wash buffer. The resin was resuspended in 800 µL of low-salt wash buffer containing 2 mg mL^–1^ 3×-FLAG peptide (ApexBio) and incubated for 30 min at 4 °C. The mixture was retrieved and centrifuged through a small filter column to remove the resin. The eluate was then loaded onto a Mono Q 5/50 GL 1 mL column on an AKTA FPLC (GE Healthcare). The column was washed with 5 mL of Buffer A (50 mM Tris-HCl, pH 8.0; 2 mM magnesium acetate; 1 mM EGTA; 1 mM DTT) and then subjected to a 26 mL linear gradient from 35–100% Buffer B mixed with Buffer A (Buffer B = 50 mM Tris-HCl, pH 8.0; 1 M potassium acetate; 2 mM magnesium acetate; 1 mM EGTA; 1 mM DTT), followed by an additional 5 mL 100% Buffer B. Fractions containing pure dynactin (~75–80% Buffer B) were pooled and buffer-exchanged through iterative rounds of dilution and concentration on a 100 kDa MWCO centrifugal filter (Amicon Ultra, Millipore) using GF150 buffer with 10% glycerol. Purity was evaluated on SDS–PAGE gels and protein aliquots were snap frozen in liquid N_2_ and stored at –80 °C.

#### Human LIS1

LIS1 constructs were purified from frozen sf9 cell pellets from a 600 mL culture, as described previously^68^. Lysis and clarification steps were similar to full-length dynein purification except lysis was performed in LIS1-lysis buffer (30 mM HEPES pH 7.4, 50 mM potassium acetate, 2 mM magnesium acetate, 1 mM EGTA, 300 mM potassium chloride, 1 mM DTT, 0.5 mM Pefabloc, 10% (vol/vol) glycerol) supplemented with cOmplete EDTA-free protease inhibitor cocktail tablet (Roche) per 50 mL was used. The clarified supernatant was incubated with 0.5 mL of IgG Sepharose 6 Fast Flow beads (GE Healthcare Life Sciences) for 2-3 hours on a roller. The beads were transferred to a gravity flow column, washed with 20 mL of LIS1-lysis buffer, 100 mL of modified TEV buffer (10 mM Tris-HCl pH 8.0, 2 mM magnesium acetate, 150 mM potassium acetate, 1 mM EGTA, 1 mM DTT, 10% (vol/vol) glycerol) supplemented with 100 mM potassium acetate, and 50 mL of modified TEV buffer. LIS1 was cleaved from IgG beads via incubation with 0.2 mg mL^–1^ TEV protease overnight on a roller. The cleaved LIS1 was filtered by centrifuging with an Ultrafree-MC VV filter (EMD Millipore) in a tabletop centrifuge. Purity was evaluated on SDS–PAGE gels, and protein aliquots were snap frozen in liquid N_2_ and stored at –80 °C.

#### Human BICD2

BICD2 construct containing N-terminal sfGFP was expressed and purified as previously described^17^. In brief, BL-21[DE3] cells (New England Biolabs) were grown at optical density at 600 nm of 0.4–0.6, and protein expression was induced with 0.1 mM IPTG for 16 h at 18 °C. Frozen cell pellet from a 2 L culture was resuspended in 60 mL of lysis buffer (30 mM HEPES pH7.4, 50 mM potassium acetate, 2 mM magnesium acetate, 1 mM EGTA, 1 mM DTT and 0.5 mM Pefabloc, 10% (vol/vol) glycerol) supplemented with cOmplete EDTA-free protease inhibitor cocktail tablet (Roche) per 50 mL and 1 mg mL^–1^ lysozyme. The resuspension was incubated on ice for 30 min and lysed by sonication. The lysate was clarified by centrifuging at 500,000*g* for 30 min in Type 70 Ti rotor (Beckman). The clarified supernatant was incubated with 2 mL of IgG Sepharose 6 Fast Flow beads (GE Healthcare Life Sciences) for 2 h on a roller. The beads were transferred into a gravity flow column, washed with 100 mL of activating-adapter-lysis buffer supplemented with 150 mM potassium acetate and 50 mL of cleavage buffer (50 mM Tris-HCl pH 8.0, 150 mM potassium acetate, 2 mM magnesium acetate, 1 mM EGTA, 1 mM DTT, 0.5 mM Pefabloc, and 10% (vol/vol) glycerol). The beads were then resuspended and incubated in 15 mL of cleavage buffer supplemented with 0.2 mg mL^–1^ TEV protease overnight on a roller. The supernatant containing cleaved protein was concentrated using a 50 kDa MWCO concentrator (EMD Millipore) to 1 mL, filtered by centrifuging with Ultrafree-MC VV filter (EMD Millipore) in a tabletop centrifuge, diluted to 2 mL in buffer A (30 mM HEPES pH 7.4, 50 mM potassium acetate, 2 mM magnesium acetate, 1 mM EGTA, 10% (vol/vol) glycerol, and 1 mM DTT), and injected into a MonoQ 5/50 GL column (GE Healthcare and Life Sciences) at 1 mL min^–1^. The column was prewashed with 10 CV of buffer A, 10 CV of buffer B (30 mM HEPES pH7.4, 1 M potassium acetate, 2 mM magnesium acetate, 1 mM EGTA, 10% (vol/vol) glycerol, and 1 mM DTT) and again with 10 CV of buffer A at 1 mL min^–1^. For elution, a linear gradient was run over 26 CV from 0–100% buffer B. The peak fractions containing sfGFP-BICD2s were collected and concentrated using a 50 kDa MWCO concentrator (EMD Millipore) to 0.2 mL. Protein was then diluted to 0.5 mL in GF150 buffer and further purified using size-exclusion chromatography on a Superose 6 Increase 10/300GL column (GE Healthcare and Life Sciences) with GF150 buffer at 0.5 mL min^–1^. The peak fractions were collected, buffer-exchanged into a GF150 buffer supplemented with 10% glycerol, concentrated to 0.2–1 mg mL^–1^ using a 50 kDa MWCO concentrator (EMD Millipore), and flash frozen in liquid nitrogen.

### TIRF microscopy

Imaging was performed with an inverted microscope (Nikon, Ti-E Eclipse) equipped with a 100×1.49 N.A. oil immersion objective (Nikon, Plano Apo). The *xy* position of the stage was controlled by ProScan linear motor stage controller (Prior). The microscope was equipped with an MLC400B laser launch (Agilent) equipped with 405 nm (30 mW), 488 nm (90 mW), 561 nm (90 mW), and 640 nm (170 mW) laser lines. The excitation and emission paths were filtered using appropriate single bandpass filter cubes (Chroma). The emitted signals were detected with an electron multiplying CCD camera (Andor Technology, iXon Ultra 888). Illumination and image acquisition was controlled by NIS Elements Advanced Research software (Nikon).

### Single-molecule motility assays

Single-molecule motility assays were performed in flow chambers using the TIRF microscopy setup described above. To reduce non-specific binding biotinylated and PEGylated coverslips (Microsurfaces) with microtubules polymerized from tubulin prepared from bovine brain, as previously described^17^. Microtubules contained ~10% biotin-tubulin to allow for attachment to streptavidin-coated coverslip and ~10% Alexa Fluor 488 (Thermo Fisher Scientific) tubulin for visualization. Imaging was done in dynein-lysis buffer supplemented with 20 µM taxol, 1 mg mL^–1^ casein, 5 mM Mg-ATP, 71.5 mM β-mercaptoethanol, and an oxygen scavenger system containing 0.4% glucose, 45 μg mL^–1^ glucose catalase (Sigma-Aldrich), and 1.15 mg mL^–1^ glucose oxidase (Sigma-Aldrich). Images were recorded every 0.3 s for 3 min. Movies showing significant drift were not analyzed.

All movies were collected by measuring TMR–dynein signal with the following protein concentrations: 83.5 pM TMR-dynein, 665 pM unlabeled dynactin, 5 nM BICD2, and 300 nM LIS1. For conditions missing LIS1, the corresponding matching buffer was used. The dynein, dynactin, and BICD2 complexes were incubated on ice for 10 min prior to LIS1 or buffer addition. Each protein mixture was then incubated on ice for an additional 10 min prior to TIRF imaging.

### TIRF motility data analysis

The velocity of moving particles was calculated from kymographs generated in Fiji as described previously^47^. Velocities were calculated only from molecules that moved processively (continuously moving along a microtubule track) for more than five frames. Non-motile or diffusive events were not considered in velocity calculations. Processive events were defined as events that move unidirectionally and do not exhibit directional changes greater than 600 nm. Diffusive events were defined as events that exhibit at least one bidirectional movement greater than 600 nm in each direction. Static events were defined as events that do not exhibit movement (less than 600 nm in each direction). Single-molecule movements that change apparent behavior (for example, shift from non-motile to processive) were considered multi-velocity events and counted as multiple events. Processive runs were calculated by counting the number of processive events for each microtubule in individual movies and dividing this number by the microtubule length. The percentage of diffusive events was calculated as diffusive events / (diffusive + stationary + processive) × 100.

### Statistical analysis

Brightness and contrast were adjusted in Fiji for all images, videos, and kymographs. All statistical tests were generated using GraphPad Prism 9. The exact value of *n*, evaluation of statistical significance, *P* values, and specific statistical analysis are described in the corresponding figures and figure legends. All TIRF experiments were analyzed from four independent replicates, and individual analysis of each replicate showed similar results. For velocity analysis, frequency distributions were first calculated for each replicate, and data were fit to a Gaussian distribution to calculate mean values. Nuclear segregation assays in Figure 4c included 8–12 biological replicates from independent cultures per condition. Statistics were generated for all biological replicates (8–12) within each condition. Nuclear segregation assay analysis for Figure 4c,i included three biological replicates from independent cultures for each condition, and statistics were generated for the three biological replicates within each condition using a one-way ANOVA with Tukey’s multiple comparisons test of each mean. Live-cell imaging experiments to assess dynein localization include three biological replicates from three independent cultures. Each replicate included >40 mid-stage mitotic cells with an *n* of at least 120 cells per condition. Each cell was assessed for the number of dynein foci at the cortex, spindle pole body or microtubule tips. Statistical analysis was performed on the means of each biological replicate using a one-way ANOVA with Tukey’s multiple comparison test.

### Reporting summary

Further information on research design is available in the Nature Portfolio Reporting Summary linked to this article.

## Online content

Any methods, additional references, Nature Portfolio reporting summaries, source data, extended data, supplementary information, acknowledgements, peer review information; details of author contributions and competing interests; and statements of data and code availability are available at 10.1038/s41594-023-01069-6.

## Supplementary information

Supplementary Information Supplementary Table 1, yeast strains

Reporting Summary

Supplementary Video 1 The structure of *S. cerevisiae* Chi (dynein–Lis1).

## Data Availability

Cryo-EM maps and molecular models have been deposited in the EM Data Bank and Protein Data Bank, respectively. Accession codes are listed here and in Table 1. Dynein^E2488Q^ bound to Lis1 in Chi conformation: EMD-27810 and PDB 8DZZ; dynein^E2488Q^ bound to Lis1 in Chi conformation, symmetry expanded: EMD-27811 and PDB 8E00. All other data will be made available upon request. Source data are provided with this paper.

## Source data

Source Data Fig. 4 Statistical Source data and analyses
Source Data Fig. 5 Statistical Source data and analyses
Source Data Extended Data Fig. 5 Unprocessed western blot
Source Data Extended Data Fig. 5 Numerical values and statistical analyses
Source Data Extended Data Fig. 6 Unprocessed SDS–PAGE gel
Source Data Extended Data Fig. 6 Numerical values and statistical analyses
