# Supplementary material for: Lis1 relieves cytoplasmic dynein-1 autoinhibition by acting as a molecular wedge
Source: Nat Struct Mol Biol. 2023 Aug 24;30(9):1357–64. doi: 10.1038/s41594-023-01069-6 (PMC10497415; doi:10.1038/s41594-023-01069-6)
Supplement: Supplementary file 1 — Supplementary Table 1, yeast strains [file 41594_2023_1069_MOESM1_ESM.pdf]

nature structural & molecular biology

Article https://doi.org/10.1038/s41594-023-01069-6

Lis1 relieves cytoplasmic dynein-1 autoinhibition by acting as a molecular wedge

In the format provided by the authors and unedited

Supplementary Table 1. *S. cerevisiae* strains used in this study.

DHA and *SNAP* refer to the HaloTag (Promega) and SNAP-tag (NEB), respectively. TEV indicates a Tev protease cleavage site. P_{GAL1} denotes the galactose promoter, which was used for inducing strong expression of Lis1 and dynein motor domain constructs. Amino acid spacers are indicated by g (glycine) and gs (glycine-serine).

Strain	Genotype	Source
RPY1	W303a (*MATa; his3-11,15; ura3-1; leu2-3,112; ade2-1; trp1-1*)	(Eshel et al., 1993)
RPY816	W303a; *pep4Δ::HIS5; prb1Δ; GAL1-8HIS-ZZ-Tev-PAC1; dyn1Δ::CgLEU2; ndl1Δ::HPH*	(Huang et al., 2012)
RPY1654	W303a; *pep4Δ::HIS5; prb1Δ; PAC11-13xMYC-TRP1; PGAL1-ZZTev-DYN1(331kDa) E2488Q; pac1Δ:: HygroR*	(DeSantis et al., 2017)
RPY1385	MATa *lys2-801 leu2-Δ1 his3-Δ200 trp1-Δ63 DYN1-3XGFP::TRP1, ura3-52::CFP-TUB1::URA3, SPC110-tdTomato::SpHIS5, ura3Δ::KanMX*	(DeSantis et al., 2017)
RPY1717	*MATa lys2-801; leu2- Δ 1; his3-Δ200; trp1-Δ63; DYN1-3XGFP::TRP1; ura3-52::CFP-TUB1::URA3; SPC110-tdTomato::SpHIS5; ura3Δ::KanMX; pac1Δ::KlURA3*	(DeSantis et al., 2017)
RPY1811	*MATa lys2-801; leu2- Δ 1; his3-Δ200; trp1-Δ63; ura3-52::CFP-TUB1::URA3; SPC110-tdTomato::SpHIS5; ura3Δ::KanMX; dyn1Δ::KlURA3*	(Gillies et al., 2022)
RPY1795	*MATa lys2-801; leu2-Δ1; his3-Δ200; trp1-Δ63; DYN1-3XGFP::TRP1; ura3-52::CFP-TUB1::URA3; SPC110-tdTomato::SpHIS5; ura3Δ::KanMX; Flag-PAC1*	(Gillies et al., 2022)
RPY1814	*MATa lys2-801; leu2-Δ1; his3-Δ200; trp1-Δ63; DYN1-3XGFP::TRP1; ura3-52::CFP-TUB1::URA3; SPC110-tdTomato::SpHIS5; ura3Δ::KanMX; Flag-pac1(W288D)*	This work
RPY1831	*MATa lys2-801; leu2-Δ1; his3-Δ200; trp1-Δ63;DYN1-3XGFP::TRP1; ura3-52::CFP-TUB1::URA3; SPC110-tdTomato::SpHIS5; ura3Δ::KanMX; Flag-pac1(N213A)*	This work
RPY1834	*MATa lys2-801; leu2-Δ1; his3-Δ200; trp1-Δ63;DYN1-3XGFP::TRP1; ura3-52::CFP-TUB1::URA3; SPC110-tdTomato::SpHIS5; ura3Δ::KanMX; Flag-pac1(N213A-W288D)*	This work
RPY1854	*MATa lys2-801; leu2- Δ 1; his3-Δ200; trp1-Δ63; DYN1(D2868K)-3XGFP::TRP1; ura3-52::CFP-TUB1::URA3; SPC110-tdTomato::SpHIS5; ura3Δ::KanMX; pac1Δ::KlURA3*	This work
RPY1861	*MATa lys2-801; leu2-Δ1; his3-Δ200; trp1-Δ63;DYN1 (D2868K)-3XGFP::TRP1; ura3-52::CFP-TUB1::URA3; SPC110-tdTomato::SpHIS5; ura3Δ::KanMX; Flag-pac1(N213A)*	This work
RPY1862	*MATa lys2-801; leu2-Δ1; his3-Δ200; trp1-Δ63; DYN1(D2868K)-3XGFP::TRP1; ura3-52::CFP-TUB1::URA3; SPC110-tdTomato::SpHIS5; ura3Δ::KanMX; Flag-pac1(W288D)*	This work
RPY1863	*MATa lys2-801; leu2-Δ1; his3-Δ200; trp1-Δ63;DYN1(D2868K)-3XGFP::TRP1; ura3-52::CFP-TUB1::URA3; SPC110-tdTomato::SpHIS5; ura3Δ::KanMX; Flag-pac1(N213A-W288D)*	This work

RPY1841	MATa *lys2-801 leu2-Δ1 his3-Δ200 trp1-Δ63 DYN1(F3446A-E3867A)-3XGFP::TRP1, ura3-52::CFP-TUB1::URA3, SPC110-tdTomato::SpHIS5, ura3Δ::KanMX*	This work
RPY1858	MATa *lys2-801 leu2-Δ1 his3-Δ200 trp1-Δ63 DYN1(D2868K-F3446A-E3867A)-3XGFP::TRP1, ura3-52::CFP-TUB1::URA3, SPC110-tdTomato::SpHIS5, ura3Δ::KanMX*	This work
