# Supplementary figures and images for: Lis1 relieves cytoplasmic dynein-1 autoinhibition by acting as a molecular wedge
Source: Nat Struct Mol Biol. 2023 Aug 24;30(9):1357–64. doi: 10.1038/s41594-023-01069-6 (PMC10497415; doi:10.1038/s41594-023-01069-6)

scan of membrane

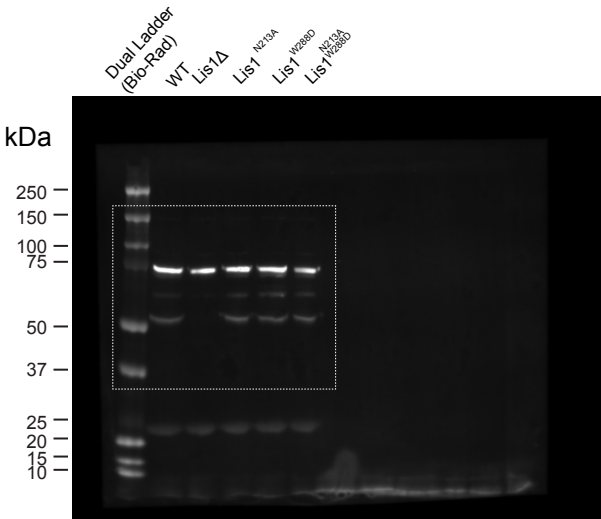

Inverted grayscale

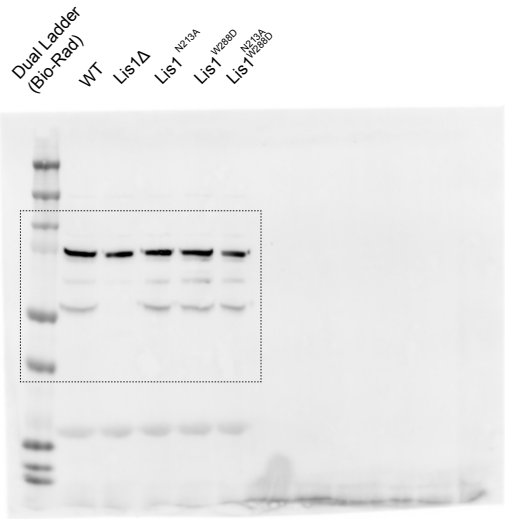

Supplement: Source Data Extended Data Fig. 5 — Unprocessed western blot [file 41594_2023_1069_MOESM6_ESM.pdf]

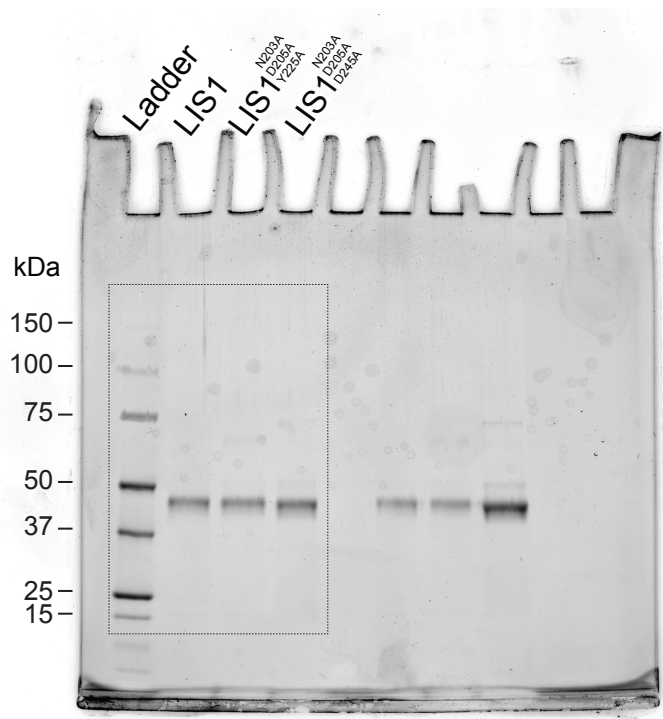

Supplement: Source Data Extended Data Fig. 6 — Unprocessed SDS–PAGE gel [file 41594_2023_1069_MOESM8_ESM.pdf]
